# Neuroprotective mechanism of syringic acid against traumatic brain injury induced cognitive dysfunction

**DOI:** 10.1002/alz.090800

**Published:** 2025-01-03

**Authors:** Rittu Banderwal

**Affiliations:** ^1^ University Institute of Pharmaceutical sciences, Panjab University, Chandigarh, Chandigarh India

## Abstract

**Background:**

Traumatic brain injury (TBI) due to external forces is a major cause of morbidity and mortality among people of all age groups, worldwide. Multiple biological processes like neuroinflammation, mitochondrial dysfunction, oxidative stress, amyloid β (Aβ) production, and tau hyperphosphorylation are involved in the pathogenesis of TBI. The role of neuroinflammation and oxidative stress has been suggested in the pathophysiology of brain injury‐induced cognitive dysfunction. Therefore, the present study was designed to explore the neuroprotective potential of syringic Acid (SA) against TBI.

**Method:**

Sprague‐Dawley male rats were exposed to brain injury using a weight drop model and kept for a rehabilitation period of 14 days after injury. Afterwards, animals were administered with SA (25 and 50 mg/kg; per oral) daily, for 14 days. Further, various behavior parameters (NOR and MWM, locomotor activity and motor coordination), biochemicals (oxidants and antioxidants markers), activities of mitochondrial enzyme complexes and acetylcholinesterase (AChE), proinflammatory markers (TNF‐α, IL‐6) were evaluated in specific brain regions.

**Result:**

Traumatized rats followed by treatment with SA (25 and 50 mg/kg, per oral) for 14 days had significantly improved memory performance (increased time spent in the target quadrant, shortened escape latency to access the platform, and increased discrimination index) in Morris water maze and novel object recognition test when compared to control (TBI) groups. Further, SA treatment significantly restored antioxidant levels, attenuated increased AChE activity, and TNF‐ α levels, and regained mitochondrial capacities. The results of the present study show that the therapeutic effect of syringic acid might involve the inhibition of inflammatory reactions against brain injury‐induced cognitive dysfunction and neuroinflammation in rats.

**Conclusion:**

The current work demonstrates the neuroprotective effect of syringic acid in an experimental model of TBI. The study further suggests that the neuroprotective impacts of syringic acid may be related to its effects on TNF‐α, IL‐6 levels, oxidative stress pathways, and mitochondrial complex capabilities.

